# Supplementary material for: Work stress and burnout among active correctional officers in Puerto Rico: A cross-sectional study
Source: PLoS One. 2024 Oct 24;19(10):e0304886. doi: 10.1371/journal.pone.0304886 (PMC11500846; doi:10.1371/journal.pone.0304886)
Supplement: Attachment — Submitted filename: PONE-D-23-15710_reviewer.pdf [file pone.0304886.s003.pdf]

PLOS ONE

“Association between work stress and burnout among active correctional officers in Puerto Rico: a cross-sectional study”

--Manuscript Draft--

**Manuscript Number:**	PONE-D-23-15710
**Article Type:**	Research Article
**Full Title:**	“Association between work stress and burnout among active correctional officers in Puerto Rico: a cross-sectional study”
**Short Title:**	Work stress and burnout in correctional officers
**Corresponding Author:**	Yaritza Diaz-Algorri, MS, DrPH<br>San Juan Bautista School of Medicine<br>Caguas, PUERTO RICO
**Keywords:**	correctional officers, Hispanics, burnout, operational stress, work stress, PSQ-Op, MBI
**Abstract:**	Introduction<br>Correctional officers (COs) are exposed to emotional and physical harm by the nature of their work. Operational stress can lead to burnout and influence absences and COs work performance.<br>Objectives<br>This study aimed to evaluate the association between work-related stress and burnout adjusted by potential confounding variables (age, sex, correctional facility, type of correctional facility, distance to work, and absenteeism).<br>Methods<br>The sample of this cross-sectional study was made up of 799 prison officials. The self-administered questionnaire consisted of four instruments: demographic data, Health and Job Performance Questionnaire, Police Operational Stress Questionnaire, and Maslach Burnout Inventory. The questionnaires were completed online and in person.<br>Results<br>A high proportion of COs reported high operational stress and burnout levels. Fatigue was the highest mean value from all stressors, with 5.89. COs reported high levels of emotional exhaustion and depersonalization. They also reported low levels of personal accomplishment. Furthermore, COs with high-stress levels are approximately eight times more likely to experience burnout.<br>Conclusion<br>These findings suggest that COs in Puerto Rico exposed to stress are more vulnerable to present burnout. The findings suggest that evidence-based interventions and programs should be implemented to help prevent and reduce operational stress and burnout among COs.
**Order of Authors:**	Lisyaima Laureano-Morales, BS, MPHc
	Nashaly Saldaña-Santiago, BS, MPHc
	Nitza Malave-Velez, BS, MPHc
	Joshua Quiles-Aponte, BS, MPHc
	Sherrilyz Travieso-Perez, BS, MPHc
	Victor Hernandez-Robles, BS, MPHc
	Yaritza Diaz-Algorri, MS, DrPH
	Alexis Vera, MPH, DBA
**Additional Information:**	
**Question**	**Response**
**Financial Disclosure**<br><br>Enter a financial disclosure statement that	The author(s) received no specific funding for this work.

Powered by Editorial Manager® and ProduXion Manager® from Aries Systems Corporation

describes the sources of funding for the work included in this submission. Review the submission guidelines for detailed requirements. View published research articles from *PLOS ONE* for specific examples.<br><br>This statement is required for submission and **will appear in the published article** if the submission is accepted. Please make sure it is accurate.<br><br>**Unfunded studies**<br>Enter: *The author(s) received no specific funding for this work.*<br><br>**Funded studies**<br>Enter a statement with the following details:<br>• Initials of the authors who received each award<br>• Grant numbers awarded to each author<br>• The full name of each funder<br>• URL of each funder website<br>• Did the sponsors or funders play any role in the study design, data collection and analysis, decision to publish, or preparation of the manuscript?<br>• **NO** - Include this sentence at the end of your statement: *The funders had no role in study design, data collection and analysis, decision to publish, or preparation of the manuscript.*<br>• **YES** - Specify the role(s) played.<br><br>* typeset	
**Competing Interests**<br><br>Use the instructions below to enter a competing interest statement for this submission. On behalf of all authors, disclose any competing interests that could be perceived to bias this work—acknowledging all financial support and any other relevant financial or non-financial competing interests.<br><br>This statement is required for submission and **will appear in the published article** if the submission is accepted. Please make sure it is accurate and that any funding	The authors have declared that no competing interests exist.

sources listed in your Funding Information later in the submission form are also declared in your Financial Disclosure statement.<br><br>View published research articles from *PLOS ONE* for specific examples.<br><br>**NO authors have competing interests**<br><br>Enter: *The authors have declared that no competing interests exist.*<br><br>**Authors with competing interests**<br><br>Enter competing interest details beginning with this statement:<br><br>*I have read the journal's policy and the authors of this manuscript have the following competing interests: [insert competing interests here]*<br><br>* typeset	
**Ethics Statement**<br><br>Enter an ethics statement for this submission. This statement is required if the study involved:<br><br>• Human participants<br>• Human specimens or tissue<br>• Vertebrate animals or cephalopods<br>• Vertebrate embryos or tissues<br>• Field research<br><br>Write "N/A" if the submission does not require an ethics statement.<br><br>General guidance is provided below. Consult the submission guidelines for detailed instructions. **Make sure that all information entered here is included in the Methods section of the manuscript.**	Human Subject Research (involving human participants and/or tissue)<br>The research project was evaluated and approved by the Institutional IRB of San Juan Bautista School of Medicine (EMSJIRB-11-2022).<br>The form of consent was written.

**Format for specific study types**<br><br>**Human Subject Research (involving human participants and/or tissue)**<br>• Give the name of the institutional review board or ethics committee that approved the study<br>• Include the approval number and/or a statement indicating approval of this research<br>• Indicate the form of consent obtained (written/oral) or the reason that consent was not obtained (e.g. the data were analyzed anonymously)<br><br>**Animal Research (involving vertebrate animals, embryos or tissues)**<br>• Provide the name of the Institutional Animal Care and Use Committee (IACUC) or other relevant ethics board that reviewed the study protocol, and indicate whether they approved this research or granted a formal waiver of ethical approval<br>• Include an approval number if one was obtained<br>• If the study involved *non-human primates*, add *additional details* about animal welfare and steps taken to ameliorate suffering<br>• If anesthesia, euthanasia, or any kind of animal sacrifice is part of the study, include briefly which substances and/or methods were applied<br><br>**Field Research**<br><br>Include the following details if this study involves the collection of plant, animal, or other materials from a natural setting:<br>• Field permit number<br>• Name of the institution or relevant body that granted permission	
**Data Availability**<br><br>Authors are required to make all data underlying the findings described fully available, without restriction, and from the time of publication. PLOS allows rare exceptions to address legal and ethical concerns. See the PLOS Data Policy and FAQ for detailed information.	Yes - all data are fully available without restriction

Powered by Editorial Manager® and ProduXion Manager® from Aries Systems Corporation

A Data Availability Statement describing where the data can be found is required at submission. Your answers to this question constitute the Data Availability Statement and **will be published in the article**, if accepted.<br><br>**Important:** Stating 'data available on request from the author' is not sufficient. If your data are only available upon request, select 'No' for the first question and explain your exceptional situation in the text box.<br><br>Do the authors confirm that all data underlying the findings described in their manuscript are fully available without restriction?	
**Describe where the data may be found in full sentences. If you are copying our sample text, replace any instances of XXX with the appropriate details.**<br><br>• If the data are **held or will be held in a public repository**, include URLs, accession numbers or DOIs. If this information will only be available after acceptance, indicate this by ticking the box below. For example: *All XXX files are available from the XXX database (accession number(s) XXX, XXX.*).<br>• If the data are all contained **within the manuscript and/or Supporting Information files**, enter the following: *All relevant data are within the manuscript and its Supporting Information files.*<br>• If neither of these applies but you are able to provide **details of access elsewhere**, with or without limitations, please do so. For example:<br><br>*Data cannot be shared publicly because of [XXX]. Data are available from the XXX Institutional Data Access / Ethics Committee (contact via XXX) for researchers who meet the criteria for access to confidential data.*<br><br>*The data underlying the results presented in the study are available from (include the name of the third party*	NA

Powered by Editorial Manager® and ProduXion Manager® from Aries Systems Corporation

and contact information or URL).<br>• This text is appropriate if the data are owned by a third party and authors do not have permission to share the data.<br><br>* typeset	
Additional data availability information:	Tick here if your circumstances are not covered by the questions above and you need the journal’s help to make your data available.

Powered by Editorial Manager® and ProduXion Manager® from Aries Systems Corporation

Manuscript
Click here to access/download;Manuscript;Manuscript BOCO May 23 2023.docx

Association between work stress and burnout among active correctional officers in Puerto Rico: a cross-sectional study.

Short title: Work stress and burnout in correctional officers

Lisyaima Laureano-Morales [1¶], Nashaly Saldaña-Santiago [1¶], Nitza Malave-Velez [1], Joshua Quiles-Aponte [1], Sherrilyz Travieso-Perez [1], Victor M. Hernandez-Robles [1], Yaritza Diaz-Algorri[*], Alexis Vera

[1]Master of Public Health Program, San Juan Bautista School of Medicine, Caguas, Puerto Rico, United States of America

*Corresponding author: Dr. Yaritza Diaz-Algorri

ydiaz@sanjuanbautista.edu

[¶]These authors contributed equally to this work.

// Abstract

Introduction

Correctional officers (COs) are exposed to emotional and physical harm by the nature of their work. Operational stress can lead to burnout and influence absences and COs work performance.

Objectives

This study aimed to evaluate the association between work-related stress and burnout adjusted by potential confounding variables (age, sex, correctional facility, type of correctional facility, distance to work, and absenteeism).

Methods

The sample of this cross-sectional study was made up of 799 prison officials. The self-administered questionnaire consisted of four instruments: demographic data, Health and Job Performance Questionnaire, Police Operational Stress Questionnaire, and Maslach Burnout Inventory. The questionnaires were completed online and in person.

Results

A high proportion of COs reported high operational stress and burnout levels. Fatigue was the highest mean value from all stressors, with 5.89. COs reported high levels of emotional exhaustion and depersonalization. They also reported low levels of personal accomplishment. Furthermore, COs with high-stress levels are approximately eight times more likely to experience burnout.

Conclusion

These findings suggest that COs in Puerto Rico exposed to stress are more vulnerable to present burnout. The findings suggest that evidence-based interventions and programs should be implemented to help prevent and reduce operational stress and burnout among COs.

Keywords: correctional officers, Hispanics, burnout, operational stress, work stress, PSQ-Op, MBI

Introduction

Studies have identified that 37% of Correctional Officers (COs) experience work stress and burnout compared to the general population with 19%. [1] COs are the people who oversee guarding, maintaining order and discipline in correctional institutions, protecting people and property, supervising and offering guidance to inmates, and contributing to their rehabilitation process.[2] Prisons present harsh and hazardous working conditions, fast rates of physical and mental fatigue, risk of infectious diseases, irregular shifts, and low financial reward rates, which systematically decrease employees' quality of life and career fulfillment.[3]

When work stress becomes chronic, it strongly affects physical and mental health; today, stress is considered a psychosocial risk in the workplace. When responses to chronic occupational stress are inadequate, burnout emerges as an occupational phenomenon, defined as "*a prolonged response to chronic emotional and interpersonal stressors*."[4] Burnout occurs in response to chronic exposure to work-related stressors and consists of three main dimensions: first, emotional burnout, defined as the expressed feelings of fatigue or chronic stress; second, depersonalization or skepticism, that is, the dimension of interpersonal context around burnout about adverse

interactions with (e.g.) supervisors, co-workers, users; and third, a significant reduction in perceptions of professional achievement in the workplace.[3] Excessive workload, insufficient staff, conflict of values, inadequate rewards, and poor work environment increase the risk of burnout.[3]

Operational stressors are related to policing specificities, such as the conditions of the correctional facility and traumatic events COs can encounter while working.[5] Work stress can become chronic and profoundly affect the physical and mental health of COs, leading them to develop exhaustion and feelings of depersonalization. The structure of correctional institutions and the relationships between management and COs can cause work stress and a feeling of dissatisfaction.[1] COs are exposed to high mental, physiological, and cognitive requirements. [6-8] This repetitive condition can lead to strain and burnout in the population. [6] Officers working in prison experience high psychological pressure due to increased job demands. [7] It has been reported that COs confront the most violent, antisocial, and problematic elements of society, putting themselves in stressful situations daily. [8] Traumatic events have been reported numerous times, including manifestations linked to stress and extreme exhaustion, known as burnout. [8]

Even though a limited number of studies have addressed the association between work-related stress and burnout in COs, none have been conducted among Puerto Rican COs. By conducting this study, we will address the following gap: data on work stress and burnout will be available regarding Puerto Rican COs. This study will evaluate the association between work-related stress and burnout adjusted by potential confounding variables (age, sex, correctional facility, type of correctional facility (maximum, minimum, and median), distance to work, and absenteeism). By better understanding these exposures and outcomes, public policy and tailored interventions can be created to improve the health and assess the needs of COs in Puerto Rico.

Methods

Sample

The study population included 3,100 COs members of the Alianza Correccional Unida (ACU) syndicate, with a sample size of n=799 COs. We calculated the sample size for this study as a convenience sample utilizing EpiInfo with a two-sided confidence level of 95% and a power of 80.0%. The ratio of unexposed (COs not exposed to work stress): to exposed (CO's that were exposed to stress) was 0.7 and calculated based on the data obtained by the literature review. A study by Hernández and Castro in 2013 reported that 27.2% (n=191 COs) of the participants in Zuera, Spain, had high levels of burnout without stress. [9] We used 27.2% as the outcome for the unexposed group. In Quebec, Canada, 37% of COs have both stress and burnout.[1] This percentage was utilized as the outcome for the exposed group.

The inclusion/exclusion criteria were: (1) being active in ACU, (2) having served as a correctional officer for more than one year, (3) having more than twenty-one years old, (4) wanting to be part of the study and signing the consent form.

Procedure, design, and Ethics

The study was performed with a cross-sectional design, in collaboration with ACU. It was promoted through email, social media, video capsules, and flyers in collaboration with ACU and its delegates. The participants accessed the questionnaire through the link in the promotions and the QR Code. The data collection stage was held from December 2022 to February 2023 through

the participants' self-reporting electronically using REDCap and on paper. The link was available for three months for the electronic self-report, and the self-report on paper was carried out during visits to Penitentiary Institutions in collaboration with ACU.

The anonymity of the participants was protected, and the fact that their participation would be used for research purposes was declared. The study design methodology was evaluated and approved by the Institutional IRB of San Juan Bautista School of Medicine (EMSJIRB-11-2022). The participants required an informed consent statement to start completing the questionnaires.

Description of questionnaires

The questionnaires were administered in Spanish and consisted of three parts. The first part of the demographic questionnaire included the variables: sex, age, civil status, annual income, years of service, correctional complex, level of security, absenteeism, burnout workshop, and distance to work.

The second section included the Police Operational Stress Questionnaire (PSQ-Op) used to assess occupational stress in police officers.[10] PSQ-Op has 20 items evaluated on a 7-point Likert scale ranging from 1 (“no stress”) to 7 (“too much stress”). The PSQ-Op has an interpretation of >3.5 high level, 2.0-3.4 moderate level, and <2.0 low level.[8] We used the validated 20-item Puerto Rican PSQ-Op with Cronbach’s alpha results of .94. [11]The third and final section included the Maslach Burnout Inventory - Human Service Survey (MBI - HSS) to measure burnout.[12] The MBI has a validated Spanish version, consisting of a 22-item 6-point scale measured by three factors (Emotional Exhaustion (EE), Depersonalization (DP), and Personal Accomplishment (PA).[12] To interpret these three factors, there is a scale that refers to the interpretation of the score obtained by participants, for PA: >=39 high level, 32-38 moderate

level, <=31 low level. DP can be measured as >=13 high level, 7-12 moderate level, <=6 low level. We measure EE as follows: >=27 high level, 17-26 moderate level, <=16 low level.[14]

Statistical Analysis

We used descriptive statistics to characterize the study population regarding socio-demographic variables. For each socio-demographic variable, the mean and standard deviation was calculated for numeric variables and frequency and percentage for categorical variables. The associations between variables were evaluated using chi-square tests for categorical variables. This study analyzed burnout as a dichotomous dependent variable (Yes burnout, No burnout). Yes burnout was considered as an outcome when a score of ≥ 27 in Emotional Exhaustion (EE) with a ≥13 in Depersonalization (DP) or ≤31 in Personal Accomplishment (PA).[13-15] To analyze the associations between operational stress and burnout, the operational stress variable was dichotomized using the median value of the PSQ total score (5.05) from which the 2 groups were created: PSQ-Op score >5.05 and PSQ-Op ≤5.05. Given the absence of literature on dichotomization and the data distribution, it was concluded that the median was the correct and ethical trend measure for dichotomization to minimize bias in the interpretation. The multivariable analysis was conducted (multiple logistic regression) to identify independent factors associated with the dependent variable burnout. Variables included in the multivariable analysis were relationship status, annual income, years of service, correctional complex, distance from work, type of facility and occupational stress. Age, sex, and absenteeism were kept in all models due to being potential confounder variables. Missing values MBI and PSQ-Op were imputed by the mode value of each instrument. While three missing values for age were imputed by the mean to execute the models of the multivariate analysis. Outliers for the distance variable were eliminated because

the data distribution was affected. Results of the associations were reported as odds ratios (OR) with 95% confidence intervals (95% CI) and p-values. All statistical analyses were conducted utilizing R version 4.2.3 and Tableau version 2022.4.1. [16-17]

Results

A total of 1,683 records from eligible participants were collected for a sample of 799 participants who completed all questionnaires. The minimum required sample was achieved with 799 COs who completed all the questionnaires. The descriptive socio-demographic statistics of the study are found in Table 1.

Using bivariate correlation analysis, Table 1 allowed us to see the measures of association and significance between the demographic variables and Burnout. After examining the association between burnout and demographic variables, no significant association was found in the following variables: years of service, annual income, age, sex, marital status, correctional complex, and security level. A significant association was found between burnout and Absenteeism with a p-value of 0.003.

Table 1. Descriptive sociodemographic characteristics of correctional officers and correlations between burnout and sociodemographic characteristics.

Variable	Description	Frequency	% or SD	p-value
Age	Mean: 43.86	796	±9.12	0.7647
Sex at birth	Male	657	82.23	0.6022
	Female	142	17.77	

Marital Status	Single	210	26.28	0.5596
	Divorce	62	7.76	
	Widowed	7	0.87	
	Married	359	44.93	
	Living Together	161	20.15	
Years of service	1 to 15	294	36.80	0.321
	16 or more	505	63.20	
Annual income	0 to $30,000	393	49.19	0.709
	$30,001 or more	406	50.81	
Correctional Complex	Northern Region	450	56.32	0.2918
	Southern Region	349	43.68	
Security level at correctional institution	Minimum	202	25.28	0.3157
	Medium	294	36.79	
	Maximum	303	37.92	
In the last 6 months, have you been absent from your duties as Correctional Officer?	Yes	544	68.0	0.003*
	No	255	31.9	
In the last 6 months, have you received any workshop or training on burnout?	Yes	20	2.50	0.5143
	No	779	97.4	
Distance from home to work (minutes)	Mean: 38.12	767	±20.80	0.5011

P values are significant at ≤ 0.05 and were obtained utilizing Chi-square
*Association is significant

Operational Stress and Burnout

The mean score value for the PSQ-Op of our study population was 4.93 (±1.93). As shown in Table 2, among COs, the highest mean value of operational stressors corresponded to "fatigue" with 5.89 (SD ±1.62). Also, as shown in Table 3, the PSQ-Op scores show that 87.10% (n=696) of the COs reported high levels of stress, 9.63% (n=77) moderate, and 3.25% (n=26) low levels of stress.

Table 2. Operational Stressors among correctional officers

PSQ-Op Item	Mean Score	SD
Shift work	5.30	1.70
Working at night alone	4.89	2.21
Over time demands	5.51	1.81
Risk of being injured on the job	5.71	1.74
Work related activities on days off (e.g., court, community events)	4.24	2.23
Traumatic events (e.g., MVA, domestics, death, injury)	4.47	2.28
Managing your social life outside of work	3.95	2.02
Not enough time available to spend with friends and family	5.43	1.81
Paperwork	4.44	1.88
Eating healthy at work	5.09	2.08
Finding time to stay in good physical condition	5.21	1.96
Fatigue (e.g., shift work, over-time)	5.88	1.62
Occupation-related health issues (e.g., back pain)	5.72	1.76
Lack of understanding from family and friends about your work	4.77	2.04
Making friends outside the job	3.99	2.15

Upholding a "higher image" in public	4.38	2.15
Negative comments from the public	4.69	2.15
Limitations to your social life (e.g., who your friends are, where you socialize)	4.33	2.13
Feeling like you are always on the job	5.51	1.83
Friends / family feel the effects of the stigma associated with your job	5.10	1.96

Table 3 Operational Stress Level Among Correctional Officers

	**Low**		**Moderate**		**High**	
	Frequency	Percent	Frequency	Percent	Frequency	Percent
**PSQ-Op Score**	26	3.25%	77	9.63%	696	87.10%

We used the MBI - HSS to measure burnout using the following scale for PA: ≥39 high levels, 32-38 moderate level, ≤31 low levels; DP: ≥13 high levels, 7-12 moderate level, ≤6 low levels, and EE: ≥27 high levels, 17-26 moderate level, ≤16 low levels. [11] Table 4 shows the mean score for the factors of burnout. EE had a mean of 37.11 (N=799, SD 13.74), with 79.10% reporting high levels of EE. The mean DP score was 15.95 (N=799, SD 8.11), with 66.83% having high DP. The mean score for PA was 28.45 (N=799, SD 10.39), with 59.95 having a low PA. Table 5 shows the results of the multiple logistic regression analysis of stress and confounding variables associated with burnout. COs who score more than 5.05 in the PSQ-Op are approximately 8 (OR 8.21, 95% CI 5.45-11.74) times more likely to experience burnout when adjusting for potential confounder variables sex, age, and absenteeism. Even though sex and age were not confounders in our study, they were included in the models for being traditional confounders in the literature.

Table. 4: Mean scores for burnout factors among correctional officers in Puerto Rico

	Low		Moderate		High	
**Factors**	Frequency	Percent	Frequency	Percent	Frequency	Percent
**Emotional Exhaustion**	87	10.89%	80	10.01%	632	79.10%
**Depersonalization**	126	15.77%	139	17.40%	534	66.83%
**Personal Accomplishment**	479	59.95%	174	21.78%	146	18.27%

Table. 5: Association between operational stress and burnout adjusted by potential confounding variables

**Models**	**n**	**OR**	**CI (95%)**
Operational stress and burnout	799	8.04	5.55,11.90
Operational stress and burnout adjusted by absenteeism	799	7.90	5.44, 11.70
Operational stress and burnout adjusted by absenteeism, sex, and age	799	8.21	5.45,11.74

Discussion

Our study responds to the call made by the scientific community to carry out studies on the prevention of burnout and work stress management [1] This study evaluated the association between work stress and burnout in active Correctional Officers. To our knowledge, this is the first research addressing this association in COs of Puerto Rico adjusted by potential confounding variables (age, sex, correctional facility, type of correctional facility (maximum, minimum, and median), distance to work, and absenteeism).

After evaluation of the demographic characteristics between burnout and COs in Puerto Rico, we found absenteeism and work stress related to the rise in burnout. The levels of emotional exhaustion and depersonalization found in this study were higher among COs from Puerto Rico than those reported in a similar study with a Hispanic population of COs from Colombia.[3] Consistent with these findings, the levels of personal accomplishment were lower for COs in Puerto Rico than those reported for Colombian COs.[3] The prevalence of burnout among COs in Puerto Rico was 70.96% which is higher than that reported in the literature for the general population (19 %) .[1] When we compare the burnout factors of COs in Puerto Rico with those of American nurses (EE=21.2, DP=5.4, PA=39.1) , the COs in this study have a higher percentage of burnout.[18] These findings suggest that COs in Puerto Rico and other places are more vulnerable to burnout than other occupations.

The PSQ-Op measures 20 factors to determine stress levels; the highest mean score reported stress factors were: fatigue (5.89), occupational-related health issues (5.72), risk of being injured on the job (5.71), over-time demand (5.51), limitations to social life (5.51) and not enough time available to spend with friends and family (5.43). Those stressors are found in the literature as some of the principal stressors in other studies like in Paleksic where fatigue (4.4), shift work (3.4), over time demands (3.6) have the highest mean, and Carleton, where fatigue (3.91) and occupation-related health issues (3.72) where part of the principal stressors.[8,19] Another important finding is that the PSQ-Op, the mean score value obtained in the study 4.93, was higher than the mean scores reported with two other studies in Canada, where the means were 3.06 and 3.31.[19-20] Even though the limited literature on a population culturally similar to the COs of Puerto Rico is important to highlight, findings may suggest that COs worldwide express similar stress levels and are affected by the same stressors.

When comparing our main association with the literature, to our knowledge, no previous study has utilized the PSQ-Op and MBI to evaluate the association between stress and burnout in COs. However, a similar study in China evaluated this association and found an association between work-related stress and burnout. This indicates that COs who report stress are more likely to have burnout than those who do not report stress.[21] Although Chinese and Puerto Rican officers are culturally different, these findings demonstrate the urgency needed to attend to the high levels of stress and burnout among COs.

Therefore, further research on managing and preventing operational stress and burnout is essential. The literature suggests that implementing evidence-based practices can decrease the levels of stress and burnout in correctional officers and other human services professionals.[22] Interventions such as mindfulness, peer-mentoring programs, and cognitive behavioral therapy have shown positive results in reducing high-stress levels and burnout.[23-25] For the proposed interventions to be more effective, the American Psychological Association recommends tailoring such interventions to gender, gender identity, culture, ethnicity, race, age, family context, religious beliefs, and sexual orientation. In the future, the authors recommend that researchers evaluate the impact of the manifestation of high-stress levels and burnout on the physical health of COs. Finally, this methodology can be replicated in other public safety personnel (police, paramedics, firefighter, and dispatchers).[19]

Strengths and Limitations

The current study had several important strengths. First, the sample size (n=799) was large enough to represent the study population. Second, the questionnaires utilized have been

psychometrically validated and are used most for the domain under study. Third, the results may directly or indirectly benefit the COs population in Puerto Rico.

Although several strengths were found, this study also had limitations that may be of direction for future studies. First, this study identified self-reported and recall biases that affected the data quality of the World Health Organization Health and Performance Questionnaire (HPQ). Based on these biases, the data collected in the HPQ could not be analyzed. Another limitation identified was that COs rotate in all 3 security levels. This restricted our ability to assess associations between each security level and burnout. Finally, we did not assess the specific type of job executed within our study population. Therefore, assessing these limitations could benefit future studies with COs.

Conclusion

This study's results indicate an association between work-related stress and burnout in COs of Puerto Rico. According to this, COs exposed to work stress are eight times more likely to present burnout, which could influence the high prevalence of burnout reported. The findings suggest that evidence-based interventions and programs should be implemented to help prevent and reduce operational stress and burnout among COs.

Acknowledgments

The authors thank Jessica Martínez, president of the United Correctional Alliance of Puerto Rico, for the main collaboration. They also thank Dr. Alexander Delgado Ramos, Dr.

Abner Vélez Vega, and Dr. Rita Vélez Alvarado for translating and facilitating the PSQ-Op. They also thank the ACU delegates, Héctor R. Travieso Miranda, Juan B. González Rivera, Sheila Santiago Rivera, Jonathan Blass Zapata, Omar Oliver Hernández, Genaro Collazo Rosario, and Byron Aquino Rodríguez, who contributed to the promotion and visits for data collection. Finally, the authors thank Dr. Yocasta Brugal, president of the San Juan Bautista School of Medicine (SJBSM) for institutional support and Dr. Estela S. Estapé, director of the SJBSM Research Center, for her review and editing of the manuscript.

Authors Contributions

1. **Conceptualization:** Lisyaima Laureano Morales, Nashaly Saldaña Santiago, Nitza Malavé Vélez, Joshua Quiles Aponte, Sherrilyz Travieso Pérez, and Victor M. Hérnandez Robles
2. **Formal Analysis:** Lisyaima Laureano Morales, Nashaly Saldaña Santiago, Nitza Malavé Vélez, Joshua Quiles Aponte, Sherrilyz Travieso Pérez, and Victor M. Hérnandez Robles
3. **Funding Acquisition:** Nashaly Saldaña Santiago, Joshua Quiles Aponte, Yaritza Díaz
4. **Investigation:**Lisyaima Laureano Morales, Nashaly Saldaña Santiago, Nitza Malavé Vélez, Joshua Quiles Aponte, Sherrilyz Travieso Pérez, and Victor M. Hérnandez Robles
5. **Methodology:** Lisyaima Laureano Morales, Nashaly Saldaña Santiago, Nitza Malavé Vélez, Joshua Quiles Aponte, Sherrilyz Travieso Pérez, and Victor M. Hérnandez Robles
6. **Project Administration:** Lisyaima Laureano Morales, Nashaly Saldaña Santiago
7. **Resources:** Lisyaima Laureano Morales, Nashaly Saldaña Santiago, Nitza Malavé Vélez, Joshua Quiles Aponte
8. **Software:** Yaritza Díaz, and Alexis Vera

9. **Supervision:** Yaritza Díaz, and Alexis Vera
10. **Validation:** Lisyaima Laureano Morales, Nashaly Saldaña Santiago, Yaritza Díaz, and Alexis Vera
11. **Visualization:** Lisyaima Laureano Morales, Nashaly Saldaña Santiago, Nitza Malavé Vélez, Joshua Quiles Aponte, Sherrilyz Travieso Pérez, and Victor M. Hérnandez Robles
12. **Writing- Original Draft Preparation:** Lisyaima Laureano Morales, Nashaly Saldaña Santiago, Nitza Malavé Vélez, Joshua Quiles Aponte, Sherrilyz Travieso Pérez, and Victor M. Hérnandez Robles
13. **Writing - Review & Editing** Lisyaima Laureano Morales, Nashaly Saldaña Santiago, Yaritza Díaz, and Alexis Vera

References

1.Finney C, Stergiopoulos E, Hensel J, Bonato S, Dewa CS. Organizational stressors associated with job stress and burnout in correctional officers: a systematic review. BMC Public Health [Internet]. 2013;13(1):82. Available from: http://dx.doi.org/10.1186/1471-2458-13-82

2. Gobierno de Puerto Rico Departamento de Rehabilitación y Corrección. Misión, visión y valores [Internet]. 2022 [cited 2022 Oct 25]. Available from: http://dcr.pr.gov/general/#:~:text=El%20Departamento%20de%20Correcci%C3%B3n%20y%20Rehabilitaci%C3%B3n%20es%20una%20organizaci%C3%B3n%20%C3%A1gil,recursos%20fiscales%20y%20reduciendo%20costos

3.Useche SA, Montoro LV, Ruiz JI, Vanegas C, Sanmartin J, Alfaro E. Workplace burnout and health issues among Colombian correctional officers. PLoS One [Internet]. 2019;14(2): e0211447. Available from: http://dx.doi.org/10.1371/journal.pone.0211447

4.Queirós C, Passos F, Bártolo A, Marques AJ, da Silva CF, Pereira A. Burnout and stress measurement in police officers: Literature review and a study with the Operational Police Stress Questionnaire. Front Psychol [Internet]. 2020; 11:587. Available from: http://dx.doi.org/10.3389/fpsyg.2020.00587

5. Queirós C, Passos F, Bártolo A, Faria S, Fonseca SM, Marques AJ, et al. Job stress, burnout and coping in police officers: Relationships and psychometric properties of the Organizational

Police Stress Questionnaire. Int J Environ Res Public Health [Internet]. 2020;17(18):6718. Available from: http://dx.doi.org/10.3390/ijerph17186718

6. Harizanova S, Stoyanova R. Burnout among nurses and correctional officers. Work [Internet]. 2020;65(1):71–7. Available from: http://dx.doi.org/10.3233/WOR-193059

7. Akbari J, Akbari R, Shakerian M, Mahaki B. Job demand-control and job stress at work: A cross-sectional study among prison staff. J Educ Health Promot [Internet]. 2017;6(1):15. Available from: http://dx.doi.org/10.4103/jehp.jehp_68_14

8. Paleksić V M. Professional stressors in prison officers: A cross-sectional study. Scr Medica [Internet]. 2020;51(3):166–73. Available from: http://dx.doi.org/10.5937/scriptamed51-24550

9. Hernandez A, Castro A. El síndrome de burnout en funcionarios del Centro Penitenciario de Zuera [Internet]. Universidad Zaragoza. Universidad Zaragoza Facultad de Ciencias Sociales y Humanas; 2013. Available from: https://Zaguán.unizar.es

10.McCreary DR, Fong I, Groll DL. Measuring policing stress meaningfully: establishing norms and cut-off values for the Operational and Organizational Police Stress Questionnaires. Police Pract Res [Internet]. 2017;18(6):612–23. Available from: http://dx.doi.org/10.1080/15614263.2017.1363965

11.Delgado Ramos, A., & Vélez Vega, A. Psychometric properties of the PSQ-OP and PSQ-ORG in Puerto Rico. Revista De Psicología. 2022 Apr;18(35).

12. Copyright ©1981 Christina Maslach & Susan E. Jackson. All rights reserved in all media. Published by Mind Garden, Inc., www.mindgarden.com

13. Lim WY, Ong J, Ong S, Hao Y, Abdullah HR, Koh DL, et al. The abbreviated Maslach Burnout Inventory can overestimate burnout: A study of anesthesiology residents. Journal of Clinical Medicine. 2019;9(1):61. doi:10.3390/jcm9010061

14. Dyrbye LN, West CP, Shanafelt TD. Defining burnout as a dichotomous variable. Journal of General Internal Medicine. 2009;24(3):440–440. doi:10.1007/s11606-008-0876-6

15. Maslach C, Jackson SE, Leiter MP. Maslach Burnout Inventory. 4th ed; 2016

16. R Core Team (2023). R: A language and environment for statistical computing. R Foundation for Statistical Computing, Vienna, Austria. URL https://www.R-project.org/

17. Tableau (version 2022.4.1). Tableau, 1621 North 34th Street, Seattle, WA 98103; info@tableau.com; http://www.tableau.com

18. Dyrbye LN, Shanafelt TD, Johnson PO, Johnson LA, Satele D, West CP. A cross-sectional study exploring the relationship between burnout, absenteeism, and job performance among American nurses. BMC Nurs [Internet]. 2019;18(1):57. Available from: http://dx.doi.org/10.1186/s12912-019-0382-7

19.Carleton RN, Afifi TO, Taillieu T, Turner S, Mason JE, Ricciardelli R, et al. Assessing the relative impact of diverse stressors among public safety personnel. International Journal of Environmental Research and Public Health. 2020;17(4):1234. doi:10.3390/ijerph17041234

20.Konyk K, Ricciardelli R, Taillieu T, Afifi TO, Groll D, Carleton RN. Assessing relative stressors and mental disorders among Canadian provincial correctional workers. International Journal of Environmental Research and Public Health. 2021;18(19):10018. doi:10.3390/ijerph181910018

21. Hu S, Wang J-N, Liu L, Wu H, Yang X, Wang Y, et al. The Association between work-related characteristics and job burnout among Chinese correctional officers: a cross-sectional survey. Public Health. 2015;129(9):1172-8.

22. Forman-Dolan J, Caggiano C, Anillo I, Kennedy TD. Burnout among professionals working in corrections: A two stage review. International Journal of Environmental Research and Public Health. 2022;19(16):9954. doi:10.3390/ijerph19169954

23. Namazi S, Kotejoshyer R, Farr D, Henning RA, Tubbs DC, Dugan AG, et al. Development and implementation of a total worker health® mentoring program in a correctional workforce. International Journal of Environmental Research and Public Health. 2021;18(16):8712. doi:10.3390/ijerph18168712

24. Kotejoshyer R, Gilmer DO, Namazi S, Farr D, Henning RA, Cherniack M. Impact of a total worker health® mentoring program in a correctional workforce. International Journal of Environmental Research and Public Health. 2021;18(16):8436. doi:10.3390/ijerph18168436

25. Alavi N, Stephenson C, Omrani M, Gerritsen C, Martin MS, Knyahnytskyi A, et al. Delivering an online cognitive behavioral therapy program to address mental health challenges faced by correctional workers and other Public Safety Personnel: Protocol (preprint). JMIR Research Protocols. 2021; doi:10.2196/30845

Click here to access/download
Supporting Information
STROBE-checklist-v4-combined-PlosMedicine.docx
