# Supplementary material for: Work stress and burnout among active correctional officers in Puerto Rico: A cross-sectional study
Source: PLoS One. 2024 Oct 24;19(10):e0304886. doi: 10.1371/journal.pone.0304886 (PMC11500846; doi:10.1371/journal.pone.0304886)
Supplement: Attachment — Submitted filename: Response to Reviewers.docx [file pone.0304886.s004.docx]

RESPONSES TO REVIEWER COMMENTS

**PLOSOne**

**Article #:** PONE-D-23-15710 R1

**Manuscript Title:** *Work stress and burnout among active correctional officers in Puerto Rico: a cross-sectional study*

**December 15, 2023**

Editor-in-Chief

Dear Dr. Emily Chenette:

Thank you for the opportunity to revise and resubmit our manuscript PONE-D-23-15710 titled *Work stress and burnout among active correctional officers in Puerto Rico: a cross-sectional study* to PLOSOne. There is a change in the title (eliminated “Association between”) according to the reviewer’s suggestion.

We thank the editor and reviewers for their time and careful consideration of our manuscript. The valuable comments and suggestions have helped us improve and clarify our paper. Responses to each reviewer's comment appear below. We also include a Table with the answers to the Editor's suggestions and describe any changes made to the manuscript. We have submitted a clean version of the revised manuscript and a version with all changes highlighted in yellow. One of the authors, Victor Hernandez, was not available for review and approval of the revised manuscript and is included in the Acknowledgments.

We look forward to your review of these revisions and hope you find this improved manuscript of value and interest to your readers.

Sincerely,

Dra. Yaritza Diaz Algorri

San Juan Bautista School of Medicine Public Health Program

PO Box 4968 Caguas PR 00726-4968

ydiaz@sanjuanbautista.edu

A. Reviewer Comments and Responses

| **Reviewer** | **Comment** | **Response** |
| --- | --- | --- |
| **1** | The study used a convenience sample, which may introduce bias and limit the generalizability of the findings. It would have been preferable to use a random sampling method to ensure a more representative sample of the population of correctional officers in Puerto Rico. | We recognize the importance of a random sample for the generalization of the data. However, a random sample would not have been entirely feasible in the selected study population. Convenience sampling was applied as it allowed easy access and adjusted to the availability of the participants. This methodology was also used by Dr. Delgado Ramos and Dr. Vélez Vega (2022) in their study. (Delgado Ramos, A., & Vélez Vega, A. Psychometric properties of the PSQ-OP and PSQ-ORG in Puerto Rico. Revista De Psicología. 2022 Apr;18 (35), 45-71).  From the beginning of the data collection stage, we observed difficulty in the participation of the COs due to the lack of free time to fill out the questionnaires. Because we could not access the institutions, we collaborated with ACU, which allowed us to have contact with the participants. Therefore, random sampling was not feasible in our situation. Convenience sampling was the strategy that allowed the viability of the study, considering the difficult access to the population. |
|  | The ratio of unexposed (COs not exposed to work stress) to exposed (COs exposed to stress) was calculated based on data obtained from the literature review. While this approach can provide some insights, it may not accurately reflect the specific context of Puerto Rico and the population of correctional officers being studied. It would have been more ideal to collect data directly from the study participants to determine the ratio of unexposed to exposed. | Regarding the ratio, there was limited information about this population and the topic of interest in the literature. In addition, the access to obtaining information about COs in Puerto Rico is difficult and limited since the information is not public. Therefore, we used data from the literature to calculate the ratios of exposed and unexposed. |
|  | The inclusion/exclusion criterion of the study is that they may limit the generalizability of the findings. | The inclusion criteria for the study were determined as follows: 1. active membership in the ACU was a primary criterion, emphasizing collaboration with ACU over the Department of Correction. Of approximately 3,200 Correctional Officers (COs), 3,100 were part of the ACU. 2. a criterion of having more than 1 year of service was established based on the PSQ-Op questionnaire, measuring operational stress in the last 6 months. This ensured that the sample had sufficient experience in the system to accurately measure operational stress. 3. individuals over 21 years of age were included based on literature and Institutional IRB requirements. This criterion aligns with previous research on the Puerto Rican population and complies with IRB regulations prohibiting the participation of minors. Finally, obtaining informed consent was a crucial criterion, following literature and IRB guidelines, mirroring the approach in other studies where the absence of signed informed consent served as an exclusion criterion. |
|  | The mentioned questionnaire administration lacks consideration for cultural and linguistic diversity. The study mentions that the questionnaires were administered in Spanish, which suggests that participants were required to have proficiency in the Spanish language. | Regarding the administration of the questionnaires in Spanish, the research protocol considered the linguistic diversity of Puerto Rico. According to the 2020 Census, 94.5% of the Puerto Rican population speaks Spanish, and only 5.4% speak English. Based on this data, we used questionnaires previously validated in Spanish in our population. |
|  | The study did not explore the potential causes of the high levels of burnout among correctional officers in Puerto Rico. While the study found a high prevalence of burnout among correctional officers in Puerto Rico and identified work stress and absenteeism as factors related to burnout, it did not investigate the underlying causes of these issues. Understanding the root causes of work stress and absenteeism could provide insights into potential interventions to reduce burnout among correctional officers. Additionally, the study did not explore the potential impact of burnout on the physical health of correctional officers, which could be an important area for future research. | We agree with the reviewer and appreciate the suggestions. Nevertheless, our study's main objective was to determine if there was a relationship between stress and burnout. Therefore, the next steps to follow in this topic will be to explore the potential causes of the high levels of burnout among correctional officers in Puerto Rico and the impact of burnout on the physical health of correctional officers. |
|  | While the study identified potential interventions to reduce stress and burnout among correctional officers, such as mindfulness, peer-mentoring programs, and cognitive behavioral therapy, it did not explore the feasibility or effectiveness of these interventions in the specific context of Puerto Rico.  The study did not explore the feasibility or effectiveness of potential interventions in the specific context of Puerto Rico. | We agree with the reviewer's comment regarding the feasibility or effectiveness of the recommended interventions. While our study did not assess the interventions mentioned in the manuscript, it's important to note that their success has been documented in CO populations and other communities in various countries. |
| **2** | In line 62, the following sentence "When work stress becomes chronic, it strongly affects physical and mental health; today, 63 stress is considered a psychosocial risk in the workplace" should have a citation. | We appreciate the recommendation and include the reference in line 62 of the manuscript. |
| **3** | We suggest eliminating the words "association between" from the title and using the following: "Work stress and burnout among active correctional officers in Puerto Rico: a cross-sectional study" | We appreciate the suggestion and the words *association between* was eliminated from the title. |
| **4** | Consider external factors that might influence burnout rates among COs in Puerto Rico. Are their political, economic, lack of personal, power outages, or sociocultural factors that could be exacerbating the issue? | We understand and appreciate the thoughtful comment about external factors. Our study focused on establishing a relationship between stress and burnout, successfully achieving its primary objectives. Recognizing the need for a comprehensive understanding, we agree that further investigations should explore the identified relationship from diverse perspectives within the population. This cross-sectional study, while exploratory in nature, served as a crucial step in addressing the existing information gap. Moving forward, we are committed to evaluate external factors beyond distance to work and civil status, aiming to provide a more comprehensive analysis of how these variables may influence burnout and work-related stress among Correctional Officers. Your valuable input will be integral to refining and expanding the scope of our research in subsequent stages. |

B. **Editor's Comments and Revisions**

| **No.** | **Comment** | **Revision** | **Location** |
| --- | --- | --- | --- |
| **'Editor's Comments** | | | |
| 1 | Citations should be “[9].”, not “.[9]” | We revised all the citations. | Lines 55-323 |
| 2 | Include the response rate and why 884 were excluded from the analysis. You could maybe include a sample flowchart. | A flowchart was created to describe the participants. | In the manuscript, the flowchart of participant recruitment will be in lines 93,107. |
| 3 | Please define what municipalities include the northern region and southern region, it is unclear. You could maybe include a map. | A map was created to describe municipalities by region. | In the manuscript, the map will be in lines 167, 182. |
| 4 | Please include if any pilot testing was conducted for the survey instruments. | No pilot study was carried out since the questionnaires were previously validated to measure the variables of interest in the population of interest. (Lines 129-134) |  |
| 5 | The authors may want to address the potential public health impacts of their findings, since burnout among COs can also affect broader societal results. | Potential public health impacts were addressed in the discussion section of the manuscript. | Lines 253-256 |
| 6 | What steps could corrections facilities take to improve support for officers and address occupational burnout? | The steps were addressed in the manuscript. | Lines 263-269 |
| 7 | The manuscript is well written. Suggestions were made to the discussion section where authors can discuss more in-depth comparisons with other studies and implications of current results. Additionally, a recommendation about cultural differences between countries compared should be provided. | We included more in-depth comparisons in the manuscript. | Lines 228-232, 247-256, 263-269 |
