# Supplementary material for: Work stress and burnout among active correctional officers in Puerto Rico: A cross-sectional study
Source: PLoS One. 2024 Oct 24;19(10):e0304886. doi: 10.1371/journal.pone.0304886 (PMC11500846; doi:10.1371/journal.pone.0304886)
Supplement: Attachment — Submitted filename: Respone Letter .docx [file pone.0304886.s005.docx]

RESPONSES TO REVIEWER COMMENTS

**April 17, 2024**

Sergio A. Useche, Ph.D.
Academic Editor
PLOS ONE

Dear Dr. Useche,

Thank you for the opportunity to revise and resubmit our manuscript PONE-D-23-15710 R2 titled “*Association between work stress and burnout among active correctional officers in Puerto Rico: a cross-sectional study*” to PLOSOne.

We want to thank the reviewers for their time and careful consideration of our manuscript. The valuable reviewer comments and suggestions have helped us improve and clarify our paper. Responses to the reviewer's comment for this second revision appears below, and any changes made to the manuscript are described. We have submitted a clean version of the revised manuscript and a version with all changes tracked.

We look forward to your review of these revisions and hope you find this improved manuscript of value and interest to your readers.

Sincerely,

Dra. Yaritza Diaz Algorri

San Juan Bautista School of Medicine Public Health Program

PO Box 4968 Caguas PR 00726-4968

ydiaz@sanjuanbautista.edu

Reviewer Comments, Generals Comment and Response Table

| **Reviewer** | **Comment** | **Response** |
| --- | --- | --- |
| **2** | While removing outliers for the distance variable is a reasonable approach to avoid skewing the data distribution, providing more context around how outliers were defined and what proportion of data was removed would allow for better assessment of the impact this preprocessing step had on the analysis results. | During the analysis process we found that some values reported for the distance to work variable were outside the established parameters, e.g. 800 minutes. Due to the data distribution being affected some values for distance were treated as outliers and eliminated. For this process we utilized the standard method Interquartile Range to identify the outliers and maintain the validity of our results. After this analysis values greater than 105 minutes were treated as outliers. Only 32 values were eliminated which correspond to 4% of this variable’s data. |

| **No.** | **Comment** | **Revision** | **Location*** |
| --- | --- | --- | --- |
| **Editor’s Comments** | | | |
| 2 | While removing outliers for the distance variable is a reasonable approach to avoid skewing the data distribution, providing more context around how outliers were defined and what proportion of data was removed would allow for better assessment of the impact this preprocessing step had on the analysis results. | We agree with the reviewer’s comment.  We have defined the outlier and methods used and we have also included the portion of data removed. | Lines 158-161 of the revised manuscript. |
